# Genetics of familial acromegaly and pituitary gigantism

**DOI:** 10.1210/clinem/dgag151

**Published:** 2026-04-08

**Authors:** Sunita M C De Sousa, Adrian F Daly

**Affiliations:** Adelaide Medical School, University of Adelaide, Adelaide 5000, Australia; Endocrine and Metabolic Unit, Royal Adelaide Hospital, Adelaide 5000, Australia; South Australian Adult Genetics Unit, Royal Adelaide Hospital, Adelaide 5000, Australia; Department of Endocrinology, Centre Hospitalier Universitaire de Liège, University of Liège, Liège 4000, Belgium

**Keywords:** pituitary, acromegaly, gigantism, genetics, familial, multiple endocrine neoplasia

## Abstract

The subset of pituitary adenomas with a heritable genetic basis is small but clinically striking. Somatotropinomas are amongst the most frequent pituitary adenoma subtypes encountered in this setting, with germline variants being enriched in familial acromegaly kindreds and people with a childhood or adolescent history of GH hypersecretion manifesting as pituitary gigantism.

The genetic causes of familial acromegaly and pituitary gigantism include variants in established pituitary adenoma predisposition genes (*AIP,* especially, but also *MEN1, CDKN1B, MAX,* and *PRKAR1A*), X-linked acrogigantism due to Xq26.3 microduplications, and McCune-Albright syndrome due to postzygotic gain-of-function *GNAS* variants. Potential associations include variants in emerging pituitary adenoma predisposition genes, including *NF1*, *PRKACB*, *PAM*, and *CHEK2*. Given the potential for gene-specific therapeutic implications in these diseases, multimodal genetic testing arranged by experienced pituitary subspecialists and conducted in expert, clinically accredited laboratories is needed to fully evaluate the genetic basis of disease. Key investigations include next-generation sequencing, chromosome microarray, and droplet digital polymerase chain reaction.

Exploratory research-based genetic testing may help uncover new genetic causes of familial acromegaly kindreds and pituitary gigantism in people with negative results on standard testing, benefiting those being tested as well as advancing our understanding of the heritable basis of somatotropinomas.

Acromegaly is a rare endocrine condition, usually caused by a growth hormone (GH)-secreting pituitary adenoma, typically presenting in the fifth decade with an approximately equal sex distribution (1, 2). Due to its insidious onset, about 70% of patients have a macroadenoma at diagnosis, but with optimal management in expert centers, adenoma size and hormonal excess can be controlled using surgery, medical treatment, and radiotherapy as individual or multimodal therapies (2).

The most striking form of acromegaly is pituitary gigantism, due to GH axis hypersecretion that begins before the closure of the growth plates. Subjects have a height > 97th percentile/ > + 2 SD above the median height for age and sex, or above final adult height in the relevant national/ethnic population (3). Pituitary gigantism has long been recognized as a specific presentation of the GH-excess spectrum, with increased morbidity (4).

Familial forms of acromegaly can present either as part of a multi-organ tumor disease, such as multiple endocrine neoplasia (MEN) syndrome, or as a condition isolated to the anterior pituitary (5, 6). The most common presentation of the latter is as familial isolated pituitary adenomas (FIPAs), defined as the occurrence of ≥2 related individuals with pituitary adenomas in the absence of other syndromic conditions (7). In FIPA kindreds, acromegaly can occur homogeneously in all affected members, or it can occur with a heterogeneous mix of other pituitary adenoma subtypes, typically prolactinomas and nonfunctioning pituitary adenomas (7). Since its initial description around 20 years ago, hundreds of FIPA kindreds have been described by large international collaborations (8, 9).

Acromegaly is typically a sporadic disease in adults due to a monoclonal expansion of differentiated somatotrophs (10). In contrast, young-onset and familial acromegaly, which account for <5% of patients with acromegaly, are forms in which germline genetic factors play an important pathophysiological role (11). Genetically determined forms of acromegaly confer unique adenoma behavior and secretory characteristics. The occurrence of GH-secreting pituitary adenomas in young individuals and families strongly points to an underlying genetic or genomic cause. Germline pathogenic *AIP* variants are the most frequent known cause of gigantism, accounting for 30% of the largest international series (3, 9). This is followed by X-linked acrogigantism (X-LAG; 10%), McCune-Albright syndrome (MAS; 5%), MEN1 syndrome (1%), Carney complex (1%), and rare individual causes (3). Many of the implicated genes are associated with both familial acromegaly and gigantism, as causes of isolated pituitary disease or as part of MEN syndromes (Table 1).

**Table 1:** Genetic causes of familial presentation of acromegaly and pituitary gigantism

Presentation	Gene and condition	Familial acromegaly	Gigantism	Molecular pathogenesis	Somatotropinoma phenotype and clinical features	Published tumor surveillance recommendations
Isolated	*AIP*; FIPA	Yes; 50% of FIPA kindreds with homogenous acromegaly have *AIP* pathogenic variants	Yes; 30% of patients with pituitary gigantism have germline *AIP* pathogenic variants	*AIP* is a tumor suppressor gene. Germline pathogenic variants/copy number variants associated with somatic “second-hit” and tumoral LOH. *AIP* inactivation leads to altered cAMP and inhibitory G-protein activity, disordered miR-34a levels, and blockade of RET-induced apoptosis.	GH-secreting and mixed GH-/prolactin-secreting adenomas; usually macroadenomas. Resistance to first-generation SRLs can occur. Multimodal therapy often required.	Marques et al 2020 (12)
	*GPR101*; X-LAG (chromosome Xq26.3 duplications)	Yes, 3 families with X-LAG	Yes; 10% of patients with pituitary gigantism have X-LAG Xq26.3 duplications	Pathogenic chromosome Xq26.3 duplications disrupt the TAD containing *GPR101* and place it under the control of ectopic enhancers, principally intronic enhancer elements at *VGLL1*. This drives GPR101 overexpression; high constitutive activity of GPR101 activates G_s_, G_q_, and G_12/13_ to drive GH and PRL excess. Hyperplasia and tumorigenesis may be driven by dysregulated GHRH secretion.	GH-secreting and mixed GH-/prolactin-secreting adenomas with or without hyperplasia; usually macroadenomas. Early childhood onset and large tumor size can make the neurosurgical approach complicated. Small tumor residues can be sufficient to maintain overgrowth and acromegaly for decades. First-generation SRLs alone are usually not effective in controlling GH/IGF-I and overgrowth; hence, pegvisomant is often necessary. Hyperprolactinemia is responsive to dopamine agonists.	Generally not applicable as the condition is completely penetrant from infancy
Syndromic	*MEN1*; MEN1	Yes; rare	Yes; 1% of gigantism	*MEN1* is a tumor suppressor gene that encodes menin, which has many interaction partners. Pathogenic germline *MEN1* variants/CNV are accompanied by a somatic “second-hit” to produce LOH.	GH-secreting and mixed GH-/prolactin-secreting adenomas. Hyperplasia is more frequently seen in MEN1-related pituitary adenomas. Clinical management similar to that of non-MEN1 related tumors.	Brandi et al 2025 (13)
	*CDKN1B;* MEN4	Yes; individual kindred	Yes; single cases	*CDKN1B is* a tumor suppressor gene that encodes p27, a protein that regulates the G1/S phase of cell cycle progression. Inactivating *CDKN1B* variants lead to delocalization of p27 from the nucleus to the cytoplasm. Low p27 expression or LOH can be seen in tumor tissue.	GH-secreting pituitary adenomas. GH-secreting pituitary tumors in MEN4 are extremely rare and do not have a defined phenotype that is different from wild-type somatotropinomas.	Wasserman et al 2025 (14)
	*MAX*; MEN5	Yes; 2 kindreds	Yes; single case	MAX is an obligate partner for the oncogene MYC, and inactivation of *MAX* leads to dysregulation across the MYC network and tumorigenesis. Germline pathogenic *MAX* variants are accompanied by a somatic second hit and LOH in the pituitary.	GH-secreting pituitary adenomas. About a dozen pituitary adenomas have been described in MEN5 patients (half had acromegaly). May occur at a younger age than wild-type acromegaly. Therapeutic characteristics are not yet well defined.	Casey et al 2024 (15)
	*PRKAR1A, PRKACB;* Carney complex	Yes	Yes; 1% of gigantism cases	*PRKAR1A* acts as a tumor suppressor gene and is accompanied by a somatic second hit and LOH.	GH- and mixed GH-/prolactin-secreting adenomas and hyperplasia. Somatotropinomas in Carney complex do not have a therapy-resistant phenotype and can be managed with standard therapies.	Stratakis 2023 (16)
	*GNAS;* McCune-Albright syndrome	No	Yes; 5% of gigantism cases	Mosaicism for post-zygotic somatic activating variants of *GNAS* (principally at Arg_201_) leads to increased cAMP and promotes pituitary hyperplasia and tumorigenesis. Such variants are not known to be inheritable.	Pituitary GH or mixed GH/prolactin hyperplasia and adenomas. Tumors can be challenging to treat due to modest responses to SRLs, and surgical access is difficult when there is concomitant skull base fibrous dysplasia. Pegvisomant can be useful in cases of poor response to SRLs. Radiotherapy is generally avoided due to the potential risk of sarcomatous transformation of dysplastic bone.	Szymczuk et al 2024 (17)

Abbreviations: cAMP, cyclic AMP; FIPA, familial isolated pituitary adenoma; GH, growth hormone; LOH, loss of heterozygosity; MEN, multiple endocrine neoplasia; PRL, prolactin; SRL, somatostatin receptor ligand; TAD, topologically associating domain; X-LAG, X-linked acrogigantism.

## Isolated presentation of familial acromegaly and gigantism

### AIP

The most frequent causes of gigantism and familial presentation of acromegaly are germline heterozygous pathogenic variants in the *AIP* gene on chromosome 11q13. *AIP* encodes a 330-amino acid protein, aryl hydrocarbon receptor (AHR)-interacting protein (AIP), which is ubiquitously expressed with a range of molecular partners across different cell types (18). Pituitary AIP is highly expressed primarily in somatotrophs and lactotrophs where it appears to co-localize with secretory granules (19). *AIP* has been considered a classical tumor suppressor gene in the pituitary gland, although in colorectal cancer and lymphoma it can also act as an oncogene (20-22). In pituitary adenomas, AIP expression occurs across all secretory cell subtypes but colocalizes with secretory granules only in GH-secreting tumors (19). AIP has several potential molecular partners (18); several of which have been described as being disrupted in the setting of pituitary tumorigenesis. Pathogenic variants in *AIP* lead to altered AIP protein expression and function in pituitary adenomas, including rapid proteasomal degradation, while others may interfere with key protein moieties (23-25). AIP inactivation leads to increased cyclic adenosine monophosphate (cAMP) in somatotroph models, which may be mediated via phosphodiesterase activity (26, 27). AIP also interacts with inhibitory G proteins, another potential mechanism that promotes tumorigenesis in the setting of pathogenic *AIP* variants (28). *AIP* inactivation leads to altered expression of microRNAs (miR), particularly miR-34a, which may constrain cell proliferation (29, 30). AIP may also form complexes with RET to induce apoptosis (31), pointing to a role for this pathway in pituitary tumorigenesis (31).

Pathogenic heterozygous *AIP* variants were first described in 2006 in large kindreds with isolated prolactinomas, somatotropinomas, and mixed somatotroph-lactotroph-secreting adenomas in Finland and Italy (20). Clinical, radiologic, and therapeutic profiles of *AIP*-related pituitary adenomas have been described (9, 19, 32, 33). Germline pathogenic *AIP* variants associate predominantly with early-onset somatotropinomas, mixed somatotroph-lactotroph adenomas, and prolactinomas, and very rarely, nonfunctioning pituitary adenomas, corticotropinomas, and thyroid-stimulating hormone-secreting adenomas. About 75% of *AIP-*related adenomas occur with acromegaly or gigantism due to GH- or mixed somatotroph-lactotroph–secreting tumors (8, 9, 33). Rare homozygous *AIP* variants are associated with a severe and fatal metabolic and cardiovascular syndrome in children (34).

Familial presentation is a major characteristic, as 68% to 85% of reported patients with *AIP*-related pituitary adenomas come from FIPA kindreds (8, 9). The remainder are “simplex” cases in which a family history of an *AIP* variant has not/cannot be established due to a lack of familial screening; true *de novo AIP* variants are reported but exceptionally rare (35).

Patients with *AIP-*related pituitary acromegaly have a significantly younger age at diagnosis than those with *AIP* wild-type acromegaly (33). Most patients present by the age of 18 years, about 2 decades earlier than those with sporadic, non-*AIP*–related acromegaly (9, 33). Another important characteristic of *AIP*-related pituitary adenomas is the large adenoma size, suggestive of an aggressive growth profile already evident at presentation (8, 9). Among 75 patients with *AIP-*related somatotropinomas, 93.1% had macroadenomas at presentation (vs 80.9% of controls with wild-type acromegaly; *P* = .023) (33). Adenomas are also more likely to expand to the cavernous sinus or toward the optic chiasma (65.1% *vs* 48.9%, respectively; *P* = .018) (33). The sex imbalance among patients with *AIP*-related pituitary adenomas, with more males affected than females (9), is driven largely by children and adolescent patients with somatotropinomas; 95% of whom are male (3).

### X-LAG

X-LAG is an ultrarare genomic disorder that leads to early-onset GH excess due to pituitary adenomas (36). X-LAG is caused by duplications at chromosome Xq26.3, leading to dysregulation of the *GPR101* gene that encodes for an orphan G protein-coupled receptor (GPCR) (37). In X-LAG, pituitary *GPR101* is overexpressed, predominantly in somatotrophs, lactotrophs, and mammosomatotrophs (36, 38). GPR101 is a highly constitutively active GPCR coupled to multiple signaling pathways (Gs, Gq, G12/13) that act together to increase GH and prolactin (PRL) secretion (39, 40). Together, this leads to chronic GH, insulin-like growth factor 1 (IGF-I), and, usually, PRL excess as early as the first months of life, driving height and weight gain beyond +2 SD before 3 years of age (41). Pituitary macroadenomas, occasionally associated with hyperplasia are typical, although GPR101 overexpression by itself does not lead to increased cell proliferation (36, 39). In some patients with X-LAG, elevated GH and PRL have been associated with moderately increased circulating growth hormone-releasing hormone (GHRH) and increased pituitary GHRH receptor levels (36, 38, 42). Because GHRH has a powerful proliferative effect on somatotrophs, this suggests the existence of hypothalamic-pituitary GHRH dysregulation in X-LAG, as no ectopic GHRH sources are present in these patients. Antagonism of GHRH in X-LAG adenoma cultures leads to decreased GH and PRL (42).

In X-LAG, tandem duplications involving *GPR101* occur due to errors during DNA replication, including fork stalling and template switching, microhomology-mediated break-induced replication, and one case induced by an *Alu-Alu* repeat (43). These small duplications in chromosome Xq26.3 alter local chromatin architecture around *GPR101*, disrupting a topologically associating domain (TAD) (37). Disease processes linked to TAD disruption are termed “TADopathies” (44). TADs are submegabase regions at which interactions between genetic elements within the TAD are more frequent than outside of the TAD (45). At the TAD boundaries, concentrations of regulatory elements include CTCF binding sites, demarcating TADs from each other. *GPR101* normally exists alone within its own TAD, and its promoter is insulated from interactions with external enhancers (37). In X-LAG, tandem duplications lead to the formation of a neo-TAD, in which *GPR101* is placed under the control of an abnormal upstream ectopic enhancer driving *GPR101* overexpression and GH hypersecretion, leading eventually to gigantism.

X-LAG usually occurs sporadically, and each individual patient has unique duplication breakpoints on chromosome Xq26.3 (36, 38, 43). In the three familial X-LAG patients described to date, the duplication was passed in an X-linked dominant manner from the affected mother to the affected son (36, 46). Most (80%) cases of X-LAG occur in females, and they have constitutive duplications at chromosome Xq26.3 and random X-chromosome inactivation (36, 38). Duplications in sporadic males with X-LAG show somatic mosaicism and have <50% of cells carrying the duplication; familial X-LAG males show an intermediate level of copy number gain (50% to 100%) (47). The reason for the sex imbalance in X-LAG is not known, but constitutive duplication may be harmful to the developing male embryo, resulting in impaired survival.

X-LAG usually presents in a choreographed manner, beginning with increased height and weight in the first year of life, rising inexorably to exceed +2 SD by 2 to 3 years of age (41). This is accompanied by large hands and feet, coarsened facial features, and widening of the interdental spaces. Other suggestive acromegaly signs and symptoms include increased appetite, headache, and acanthosis nigricans, and signs of precocious puberty (41). GH is always increased, sometimes to extremely high levels, and IGF-I concentrations are above the appropriate upper limit of normal for age and sex. Hyperprolactinemia is also usually present at diagnosis, and similar to GH, elevations in baseline PRL can be very high (36, 43, 48, 49). When present, GHRH excess is modest in X-LAG and lower than seen with ectopic GHRH-secreting neuroendocrine tumors. Pituitary magnetic resonance imaging (MRI) in X-LAG, crucial for completing the diagnostic phase and for neurosurgical referral, typically shows a pituitary macroadenoma (median maximal diameter, 18.2 mm); images consistent with hyperplasia also are seen, and in one patient a microadenoma was diagnosed (41). A single patient with clinical X-LAG with an Xq26.3 duplication had increased GH and IGF-I secretion, physical overgrowth, but no convincing evidence of hyperplasia/adenoma on MRI during long-term follow-up (49).

Management of X-LAG, similar to other forms of pituitary gigantism, relies on early diagnosis and effective hormonal control to reduce GH and IGF-I and to maintain final height as near to the normal range as possible (4). Patients with X-LAG are the youngest group affected by adenomatous GH excess, which raises therapeutic challenges. Neurosurgical resection of a macroadenoma in a young patient can be challenging due to the immature cranium and skull base, which can limit access. Also, adenomatous and hyperplastic tissue in X-LAG can be extensive and difficult to differentiate from healthy pituitary. X-LAG is unusual in that very small amounts of residual adenoma, sometimes not visualized on MRI, can remain active even after extensive surgical resection, and may require decades of therapy to suppress chronic GH and IGF-I excess (41). In X-LAG with extensive hyperplastic involvement, anterior hypophysectomy has been used to definitively control hormonally driven overgrowth (36).

When used alone as medical therapy in X-LAG, somatostatin receptor ligands (SRLs) are insufficient to control GH and IGF-I, even at adult dosages in very young children. Following gross total resection, adjuvant SRLs at adult dosages can further inhibit excess GH/IGF-I, but hormonal control is rarely achieved (48). The GH receptor antagonist pegvisomant may reduce IGF-I in X-LAG when used alone and in combination with SRLs (38). Hyperprolactinemia, when present, is rapidly controlled with low to moderate doses of dopamine agonists. Owing to the slow onset of action, radiotherapy is not a major therapeutic option to produce the rapid hormonal control and reduction of height gain that is necessary in X-LAG (38, 43).

Most patients with X-LAG require multimodal therapy to control GH hypersecretion and skeletal overgrowth. High secretory activity of residual adenoma tissue requires extensive anterior pituitary resection, and hypophysectomy may sometimes be required. The cumulative use of surgery, radiotherapy, and medical therapies engenders hypopituitarism as a frequent cost of hormonal control in X-LAG (41).

## Syndromic presentation of familial acromegaly and gigantism

### MEN1

MEN type 1 (MEN1) is an autosomal dominant disease caused by heterozygous pathogenic variants in *MEN1*, a tumor suppressor gene located on chromosome 11q13 (50). *MEN1* encodes menin, a 660-amino acid protein that is involved in the regulation of multiple cell processes. MEN1 is characterized classically by neoplasia affecting the parathyroids, endocrine pancreas, and pituitary, but is associated with tumors in other endocrine and nonendocrine tissues. MEN1 has a high penetrance in adulthood, driven mainly by parathyroid disease. Pituitary adenomas occur in about 36% to 52% of individuals with MEN1 (13). Early work suggested that pituitary adenomas in MEN1 were more aggressive than wild-type matched controls (46). Over the past 20 years, the epidemiology of pituitary adenomas in MEN1 has changed due to MRI screening programs leading to the identification of microadenomas in individuals with MEN1, many of which do not progress (51). The new 2025 MEN1 guidelines consider that the type and clinical behavior of MEN1-associated pituitary adenomas are generally similar to wild-type (13). However, as the guidelines note, patients with germline heterozygous *MEN1* can occasionally present with young-onset and aggressive pituitary adenomas (13, 52).

Acromegaly and gigantism account for 4% to 9% of patients with pituitary adenoma due to MEN1 (13). Usually, the presentation and management of acromegaly in patients with MEN1 does not differ from those without MEN1 and should follow the Pituitary Society guidelines (53).

### MEN4

MEN type 4 (MEN4) is caused by pathogenic germline *CDKN1B* gene variants, shares clinical similarities with MEN1, but is much rarer (54). In an extensive multiyear screening program in France, pathogenic *CDKN1B* variants were identified in 0.07% of 5600 individuals undergoing genetic studies for suspected MEN syndromes (55). While 25% of patients with MEN4 develop pituitary adenomas, only ∼30 cases of pituitary adenomas have been reported (54, 56-58). Secreting and nonfunctioning clinical subtypes range from small microadenomas to invasive macroadenomas (56, 59-61). Fewer than 10 patients with acromegaly have been described in association with germline *CDKN1B* variants in MEN4. The original MEN4 kindred had familial acromegaly in two members, underscoring that it represents a very rare form of familial pituitary disease (54). Pediatric-onset acromegaly-gigantism in the setting of *CDKN1B* is limited to case reports (62).

### MEN5

MEN type 5 (MEN5) is a new MEN syndrome, and its clinical phenotype is evolving quickly. MEN5 is caused by germline pathogenic variants in the *MAX* gene, a key molecular partner for the oncogene *MYC*; together with its proximal network of partners, *MYC* is one of the most commonly altered cancer genes (63, 64). *MAX* was first implicated in the pathogenesis of hereditary and sporadic pheochromocytomas and paragangliomas (65-67). In parallel, the number of other endocrine and nonendocrine organs affected by *MAX*-related tumors has grown. About 11 patients with pituitary adenomas and MEN5 have been described, most of whom also had pheochromocytomas (68-72). Germline *MAX* pathogenic variants have been identified in teenagers and a single patient with pituitary gigantism (69, 73). An overt familial acromegaly kindred with MEN5 has been reported, and an Australian family with MEN5 had a member with acromegaly and another with elevated IGF-I and a bulky pituitary (68, 69).

### Carney Complex

Carney complex, due to germline pathogenic variants in the *PRKAR1A* gene, has a classical triad consisting of cardiac myxomas, spotty skin pigmentation, and endocrine overactivity, with the latter including adrenal, pituitary, and other tumors (74). Disease activity is driven by constitutive overactivity of protein kinase A in affected tissues, leading to increased cAMP levels. Pituitary disease in Carney complex can evolve from biochemical GH excess without visible adenoma on MRI, to mammosomatotroph hyperplasia and later adenomas (commonly microadenomas) (75-77). Regular screening has identified a clinical phenotype in which mild or fluctuating GH/IGF-I excess can give rise to overt disease over time (76-79), leading to an increased prevalence of acromegaly (mild and overt) from 10% to nearer 20%. Acromegaly in Carney complex is usually diagnosed when patients are in their 30s, and pediatric-onset disease or pituitary gigantism are very rare (3, 77).

*PRKACB* (chromosome 1p31.1, OMIM *176892) encodes protein kinase cAMP-activated catalytic subunit beta (PRKACB) and has been implicated in Carney complex. Whereas LOF *PRKAR1A* variants result in inactivation of one of the regulatory subunits of protein kinase A and hence increased protein kinase A activity, gain-of-function *PRKACB* variants result in activation of the corresponding catalytic subunit that may cause increased protein kinase A activity. Germline triplication of the *PRKACB* locus was reported in a young woman with *PRKAR1A* wild-type Carney complex, including pituitary gigantism (height >97th percentile) that was successfully managed with transsphenoidal resection of the histologically proven somatotropinoma (80). However, array comparative genomic hybridization (aCGH) testing for chromosome 1p31 amplifications among an additional 40 patients with *PRKAR1A* wild-type Carney complex was negative (80). A subsequent study of *PRKACB* in 148 subjects with primary pigmented nodular adrenocortical disease and other protein kinase A–mediated conditions, but without *PRKAR1A* defects, found two patients with possibly pathogenic *PRKACB* variants (81). Overall, *PRKACB* variants appear to be an exceptionally rare cause of endocrine tumorigenesis.

### McCune–Albright syndrome

McCune–Albright Syndrome (MAS) was originally described in 1937 and consists of a triad of bony lesions (polyostotic fibrous dysplasia), characteristic *café-au-lait* macules, and endocrine disorders (82). The most common endocrine disorders include precocious puberty, GH excess, Cushing's syndrome, and hyperthyroidism. MAS is rare, caused by postzygotic mosaicism for an activating pathogenic variant (usually at arginine 201, rarely at glutamine 227) in the *GNAS* gene, which leads to constitutive activation of the α subunit of the G protein and elevated cAMP and overgrowth/overactivity of affected tissues (83). MAS is not familial, and therefore, kindreds are not seen. MAS is a recognized cause of overgrowth in childhood due to precocious puberty or early-onset GH excess. The former eventually is associated with decreased final height if premature growth plate closure occurs. MAS is a well-recognized cause of pituitary gigantism, accounting for about 5% of known cases (4). GH and IGF-I excess in MAS is challenging as it occurs early in life and can be due to diffuse anterior pituitary hyperplasia, which is difficult to treat surgically. Hyperprolactinemia regularly accompanies GH excess (82). In addition, GH excess can coexist with craniofacial fibrous dysplasia, and the interaction between these can result in significant cranial and skull base deformity, compromising cranial nerves and further complicating surgical access (84-86). Given the diffuse anterior pituitary involvement by adenoma and/or hyperplasia, a definitive surgical cure is difficult without extensive or total anterior hypophysectomy (82, 87, 88). Medical therapy for acromegaly and gigantism in MAS can require multimodal therapy as SRL resistance can occur, and GH receptor antagonist therapy may normalize IGF-I levels (82, 84, 87).

### *SDHx* genes

The *SDHx* genes (*SDHA*, *SDHB*, *SDHC*, and *SDHD*) each encode a subunit of succinate dehydrogenase, an enzymatic complex that converts succinate to fumarate in the Krebs cycle and transfers electrons from succinate to the electron transport chain. The *SDHx* genes function as tumor suppressor genes, with *SDHx* variants resulting in pseudohypoxia and consequently a tumorigenic environment. Germline loss-of-function (LOF) *SDHx* variants, most established in their role in pheochromocytoma/paraganglioma development, have been observed with pituitary adenomas. When combined with pheochromocytoma/paraganglioma, the condition is referred to as the 3P (pheochromocytoma, paraganglioma, and pituitary adenoma) association syndrome (89, 90). *SDHx*-associated pituitary adenomas are rare, but when they occur, somatotropinoma with a positive family history for pituitary adenomas (prolactinoma) can occur (90). To the best of our knowledge, *SDHx*-related cases of familial acromegaly or pituitary gigantism have not been reported.

## Rare and emerging genetic causes

Germline variants in *NF1*, *TSC2,*  *PAM*, and *CHEK2* have been reported occasionally in association with GH excess and are hence considered candidate genes in the pathogenesis of familial acromegaly and pituitary gigantism. *NF1* (chromosome 17q11.2, OMIM *613113) encodes neurofibromin, a cytoplasmic protein primarily expressed in neurons, Schwann cells, oligodendrocytes, and leukocytes, and is involved in the regulation of the mitogen-activated protein kinase/extracellular signal-regulated kinase (MAPK/ERK) pathway, adenylyl cyclase function, and cytoskeleton assembly (91). Germline LOF variants produce neurofibromatosis type 1 (NF1), a progressive autosomal dominant multiple neoplasia syndrome that occasionally includes GH excess of variable etiology and is reported in association with somatotropinomas or NF1-related optic pathway gliomas with either a normal or enlarged pituitary (92). The mechanism of GH excess in optic pathway glioma is postulated to be loss of somatostatin-mediated inhibition of GH caused by glioma infiltration of the hypothalamic-pituitary region (93). It is plausible that the presumed somatotroph hyperplastic effect of reduced somatostatin may ultimately unleash tumorigenesis and account for somatotropinoma formation, although NF1-related somatotropinomas have occurred in the absence of optic pathway glioma (92). However, somatic studies of NF1-related pituitary adenomas are limited, and *NF1* loss of heterozygosity (LOH) has not been identified (94). Since *NF1* is a tumor suppressor gene, the lack of LOH argues against *NF1* variants as a direct cause of somatotropinomas, although LOH is not invariable, even in classic NF1-related tumors (95). Standard pituitary adenoma management is generally appropriate in NF1-related somatotropinomas (92), although radiotherapy should be avoided unless essential, given the heightened risk of second CNS tumors observed in patients with NF1-related optic pathway gliomas who are treated with radiotherapy compared to their nonirradiated counterparts (96). In children with GH excess due to NF1-related optic pathway gliomas, SRLs may be used to normalize IGF-I and growth velocity (97).

*TSC2* (chromosome 16p13.3, OMIM *191092) encodes the growth inhibitory protein, tuberin, which interacts with hamartin (encoded by *TSC1*) to form the tuberous sclerosis complex (TSC) protein complex. Germline LOF variants produce TSC, an autosomal dominant multisystem condition manifesting as widespread hamartomas (96). A historical case report described a child with TSC and gigantism (98) while the Eker rat model of TSC due to a spontaneous germline variant in the *Tsc2* gene demonstrates pituitary adenomas in nearly 60% of adult rats with frequent LOH in these adenomas (99, 100). However, despite CNS imaging for tumor surveillance in TSC, pituitary adenomas are isolated, suggesting that the association in humans may be coincidental rather than causative (96).

*PAM* (chromosome 5q21.1, OMIM *170270) encodes peptidylglycine alpha-amidating monooxygenase, the sole enzyme responsible for C-terminal amidation of peptides, including peptide hormones (98). *PAM* is a candidate pituitary adenoma predisposition gene, with germline variants implicated in hypersecretory adenomas, including sporadic and familial acromegaly and pituitary gigantism (101, 102). How *PAM* variants might induce somatotroph tumorigenesis is currently unclear. A multi-cohort study of Swedish and UK populations found that two missense variants (S539W and D563G, previously identified in somatotropinomas) are associated with reduced amidating activity and increased GH and IGF-I levels, albeit with a phenotype (shorter stature, lower body weight, and reduced muscle and bone mass and muscle strength) akin to GH deficiency rather than excess (103). Further clinical and basic research is required to elucidate the role of *PAM* in somatotroph function and neoplastic transformation.

*CHEK2* encodes checkpoint kinase 2, a protein kinase activated in response to DNA damage and that induces cell cycle arrest. *CHEK2* is a moderate penetrance cancer predisposition gene that is unequivocally associated with breast cancer and possibly associated with colorectal and prostate cancer, amongst a variety of other neoplasms (104). Similar to *PAM*, germline *CHEK2* variants are enriched in the pituitary adenoma setting and associated with a variety of pituitary adenoma subtypes, including somatotropinomas (105, 106). This recently described gene-disease relationship is under further investigation through collaborative cohort and functional studies aimed at delineating the specific effects of *CHEK2* variants on adenohypophyseal cells.

Somatic variants are associated with sporadic adenoma formation and thus are not relevant in familial acromegaly, but somatic variants could play a role in pituitary gigantism through sporadic childhood-onset somatotropinomas. Somatic genetic and molecular changes found in somatotropinomas include gain-of-function *GNAS* variants as driver mutations underlying sporadic acromegaly (107). More recently, loss of AHR (a binding partner of AIP) and KDM1A (a regulator of gene expression via histone demethylation) have emerged (25, 108, 109). Germline *GNAS* variants are lethal and hence not a contributor to familial acromegaly, but pituitary gigantism may arise through childhood-onset sporadic somatotropinoma formation due to somatic *GNAS* variants or somatotroph hyperplasia/neoplasia through the postzygotic *GNAS* variants underlying MAS. Emerging pituitary adenoma predisposition genes have not yet been established to play a specific role in the somatotroph lineage, but may become apparent in time with ongoing genomic studies of people with familial acromegaly or pituitary gigantism (110).

## Treatment implications in familial acromegaly and pituitary gigantism

The clinical management of patients with familial acromegaly and pituitary gigantism should follow acromegaly guidelines (111). The major genetic contributors to familial acromegaly and pituitary gigantism may impact the clinical presentation and treatment of somatotropinomas with some challenging therapeutic profiles. The first important factor is the younger age at diagnosis for pituitary gigantism. The macroadenomas usually seen in association with *AIP* germline variants and in X-LAG may present further neurosurgical challenges, especially with an immature parasellar region. Pathogenic *AIP* variants and X-LAG are also associated with high GH/IGF-I secretion that is relatively resistant to SRL therapy. Use of pasireotide or pegvisomant can be made on a case-by-case basis. PRL cosecretion is often severe enough in X-LAG to warrant effective dopamine agonist therapy. In MEN1, MEN4, MEN5, and Carney Complex, responses to surgical and medical therapies are closer to those generally seen in sporadic acromegaly. A specific treatment goal for patients with incipient pituitary gigantism is to limit final adult height as close as possible to predicted height. Hence, time is a crucial factor in these patients and practical efforts should be made to undertake timely referrals for investigations and to achieve effective IGF-I control as soon as possible. Thus, the slow-onset therapeutic effects of pituitary irradiation are unlikely to be adequate alone to control pituitary GH excess in young individuals. Concerns about the malignant transformation of pathologically hypertrophic bone tissue have limited the use of radiotherapy in MAS.

## Approach to genetic testing in familial acromegaly and pituitary gigantism

Germline genetic testing should generally be considered in all people with familial acromegaly or pituitary gigantism. The rationale for testing in these settings is that familial disease and pediatric-onset disease are the two key risk factors for germline variants in pituitary adenoma predisposition genes; somatotropinomas are among the pituitary adenoma lineages most commonly associated with identifiable genetic causes; and finding a germline genetic cause for pituitary adenomas can guide the intensity of management, gene-specific surveillance for other relevant tumors, reproductive planning, and cascade testing of at-risk relatives (112). Although somatic genetic testing is gaining traction, particularly for aggressive pituitary adenomas, to guide tumor-targeted therapies, the specific somatic genes that have thus been recommended for testing (*TP53*, *ATRX*, and *SF3B1*) are not yet implicated in somatotropinoma pathogenesis and are not relevant here (113).

**Figure 1:**
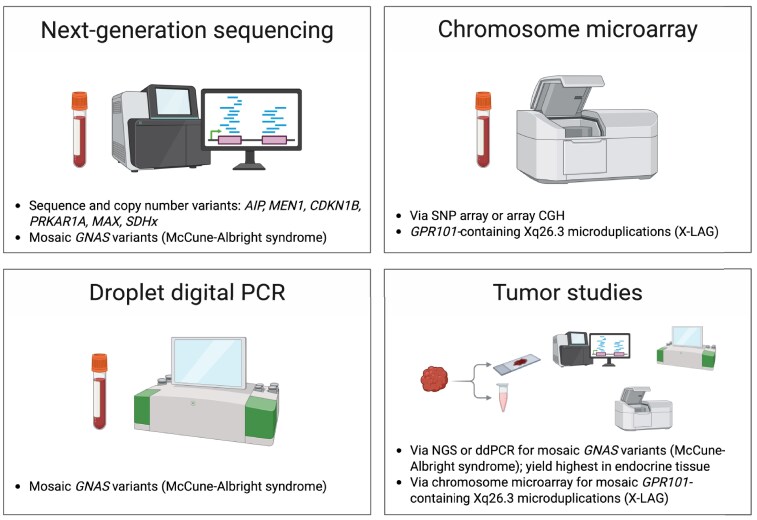
Genetic testing methodologies in somatotropinomas. The clinically available tests used in the genetic assessment of somatotropinomas are described, including the key diagnoses identified with these modalities. Mosaicism may be found in *GNAS* (producing McCune-Albright syndrome), Xq26.3 (producing X-LAG), or any of the other pituitary adenoma predisposition genes. Abbreviations: CGH, comparative genomic hybridization; ddPCR, droplet digital polymerase chain reaction; NGS, next-generation sequencing; PCR, polymerase chain reaction; SNP, single-nucleotide polymorphism; X-LAG, X-linked acrogigantism. Figure created with BioRender (https://www.BioRender.com/6ukgzub).

The general clinical approach to endocrine genetic testing should include pre- and post-test counseling and result interpretation (114). In familial acromegaly and pituitary gigantism, genetic testing should ideally be requested by clinical teams with collective subspecialty expertise in pituitary adenomas and clinical genetics, following the principles of the Pituitary Society Pituitary Tumor Centers of Excellence framework, which governs other areas of pituitary adenoma practice (115). The wide range of potential underlying genetic causes of familial acromegaly and pituitary gigantism necessitates a multimodal test repertoire, including gene panel testing, chromosome microarray (CMA) or analogous tests, and dedicated tests for mosaicism (Fig. 1), which should be undertaken in expert, clinically accredited laboratories. The required germline DNA may be extracted from peripheral blood, saliva, or buccal swab specimens (114). The latter is often preferred in young children, although children with pituitary gigantism typically have regular blood collections for hormone monitoring, and genetic testing may be scheduled to coincide with biochemical testing.

With the exceptions of X-LAG and MAS addressed below, germline gene panel testing via next-generation sequencing (NGS) enables simultaneous and comprehensive assessment of the relevant genes in familial acromegaly or pituitary gigantism. NGS panel testing may be a targeted gene panel, with which all genes of interest are sequenced and analyzed, or a virtual gene panel, where all genes are sequenced on a larger, whole-exome, or genome sequencing backbone but only genes of interest are analyzed (114). The inherent advantage of the latter approach is the ability to re-analyze raw sequencing data as new predisposition genes are discovered. Such re-analyses have proven fruitful in the research setting (102, 105). Clinical gene panel testing in familial acromegaly or pituitary gigantism should include analysis of all established genes (*AIP*, *MEN1*, *CDKN1B*, *PRKAR1A*, *MAX*, and *SDHx*) to maximize testing yield but not emerging genes (eg, *PAM*, *CHEK2*) to minimize the burden of variants of uncertain significance (VUS). However, a clinical phenotype of NF1 or *PRKAR1A*-negative Carney complex in patients with GH excess may warrant *NF1* or *PRKACB* testing, respectively. Accredited NGS calling pipelines for sequence and copy number variants (CNVs) should be used, and variants should be reported in accordance with the American College of Medical Genetics and Genomics variant classification framework, with pathogenic and likely pathogenic constituting “positive” results and VUS regarded as “uninformative” (116). CNV detection is an important component of comprehensive germline genetic testing as causative germline CNVs have been described in pituitary adenoma predisposition genes and account for subsets of MEN1 syndrome, Carney complex, among others (16, 117).

X-LAG requires CMA to identify the causative Xq26.3 microduplications. CMA may be performed by either single-nucleotide polymorphism array or array CGH technologies. Single-nucleotide polymorphism array is more expensive and offers higher resolution, but the typical commercially available array CGH platform with 60 000 probes is sufficient to identify the Xq26.3 microduplications, which are typically about 600 kilobase (37). Despite these considerations, the choice of microarray is typically governed by the technology offered by laboratories, which tend to offer either one or the other technology rather than both. As recently demonstrated through clinical, genomic (RNA sequencing, assay for transposase-accessible chromatin using sequencing, and H3K27ac chromatin immunoprecipitation sequencing), and bioinformatic studies, currently described pathogenic microduplications in X-LAG appear to rely on ectopic enhancers in *VGLL1* to drive *GPR101* misexpression, although the precise roles of these and other potential ectopic enhancers remain to be validated experimentally (37, 118-120). While techniques to assess chromatin conformation, such as 4C/HiC, are the gold standard research tools for exploring TAD disruptions (120), they are costly and labor-intensive approaches and currently are not readily deployable to diagnostic laboratories (118, 119). *In silico* predictors based on normal and pathogenic chromatin structure promise to facilitate rapid assessment of the pathogenicity of TAD disruptions (119).

Mosaic *GNAS* variants underlying MAS may be difficult to detect depending on allele fractions in a given person and the supplied specimen. Because the disorder is never familial and typically presents with a classic multisystem phenotype, genetic testing often is not additive to patient care but may be helpful to confirm a diagnosis of MAS in less compelling presentations—for example, pituitary gigantism with no or minimal fibrous dysplasia and no other endocrine manifestations to date (114). If genetic testing is required, NGS is particularly preferred over Sanger sequencing as it allows for visualization of variants at allele frequencies well below 0.5 (121). NGS of tissues of different lineages (eg, blood and resected tumor specimens) may increase the likelihood of detecting mosaic *GNAS* variants. Historical data from polymerase chain reaction testing have shown that *GNAS* variant detection is highest in endocrine tissues and lowest in affected skin specimens, with intermediate detection rates in blood; therefore, skin biopsies of café-au-lait macules may be of low yield, whereas testing of resected glandular tissue may be revealing when peripheral blood testing is negative (122). Droplet digital polymerase chain reaction is another option to detect mosaicism—albeit not widely available—that involves high-resolution detection of allele fractions (121-124). These principles of mosaicism detection also should be considered in people with a highly compelling phenotype for either X-LAG or one of the other genes implicated in familial acromegaly and pituitary gigantism but with negative results on germline CMA or gene panel testing, respectively, as mosaic Xq26.3 microduplications and mosaic variants in pituitary adenoma predisposition genes have been well described (47, 125).

Research-based testing may be considered in patients with especially compelling phenotypes for an underlying genetic cause, but with negative results on standard genetic testing. This is especially important when there is a high clinical utility of identifying the precise molecular cause—for example, to guide cascade testing in multiple neoplasia conditions to rationalize tumor surveillance in family members or to facilitate *in vitro* fertilization with preimplantation genetic testing for a childhood-onset condition. In the exploratory setting, genetic testing may involve re-assessment of VUS, including targeted analysis of putative mechanisms such as RNA studies to confirm aberrant splicing, intronic sequencing to identify occult variants in known predisposition genes, and/or exome or genome sequencing to identify variants in emerging or new pituitary adenoma predisposition genes.

## Management of positive genetic test results

Apart from McCune-Albright syndrome, which is noninherited, cascade testing should be offered to clinically affected family members of patients with identified germline pathogenic variants to confirm the variant as the cause of familial acromegaly/gigantism and guide healthcare of these relatives. Cascade testing should also be offered to clinically unaffected family members to facilitate surveillance in those who test positive for the familial variant. An exception to this is X-LAG: as a fully penetrant condition beginning in infancy, noninfant unaffected relatives are not expected to have inherited the causative Xq26.3 microduplication, and thus, cascade testing is generally not warranted in this group.

Cascade testing may be performed via single amplicon-based Sanger sequencing, as the sequence variant is known and interrogation of the whole gene or other genes is not required. However, an NGS approach is now preferred for cascade testing by some laboratories due to economies of scale provided by NGS use throughout a laboratory compared to the custom amplicon design required for Sanger sequencing. Cascade testing for CNVs may be performed either via NGS—provided the pipeline utilized is predicted to identify the specific CNV—or MLPA. In the rare event that cascade testing is performed for X-LAG, this should be performed via CMA.

Surveillance should be offered to families with an identified germline variant. For *AIP,* pituitary adenoma surveillance is recommended in variant-positive clinically unaffected relatives, whereas the proband and relatives already known to have pituitary adenomas do not require additional surveillance, as *AIP* variants are not associated with an increased risk of extrapituitary neoplasms. Pituitary adenoma penetrance is relatively low, with only 15-30% of *AIP* heterozygotes developing pituitary adenomas (9, 20, 32, 126) (approx. 80% of these are associated with GH hypersecretion (12)). Nonetheless, pituitary adenoma surveillance is indicated in clinically unaffected individuals found to have an *AIP* pathogenic variant, as *AIP* heterozygotes with surveillance-detected pituitary adenomas have smaller and less invasive adenomas and require less intense pituitary adenoma management compared to clinically presenting counterparts (12). Although there are currently no international consensus guidelines governing pituitary adenoma surveillance in people with *AIP* pathogenic variants, a reasonable approach is to perform annual clinical assessment and IGF-I and PRL measurement from the time of variant detection, in addition to 5-yearly pituitary MRI from the time of variant detection or 10 years of age (whichever is older). As most *AIP* heterozygotes with pituitary adenomas clinically manifest by age 20 years, with no *AIP* heterozygotes reported to develop new-onset pituitary adenomas following normal pituitary assessment at age 30 years or later, surveillance frequency may be relaxed between ages 21 and 30 years and then ceased (8, 12).

There is generally no indication for *GPR101*-guided tumor surveillance because *GPR101*-containing X chromosome microduplications are associated only with pituitary tumorigenesis, and penetrance is complete and begins in infancy. Accordingly, there have been no reports of unaffected carriers of truly pathogenic *GPR101*-containing X chromosome microduplications. A theoretical exception to this would be apparently unaffected infant relatives of individuals with X-LAG who may undergo cascade testing for familial *GPR101*-containing X chromosome microduplication and then, if positive, require close monitoring of length and weight centiles which would be expected to escalate quickly as the disorder manifests.

Variants in genes related to syndromic presentations should prompt surveillance for all relevant tumors not already detected in the proband and their variant-positive relatives. Such surveillance should follow the latest international consensus guidelines where available, or otherwise gene-specific literature (see Table 1).

Finally, in both probands and relatives, knowledge of variant status may additionally guide reproductive planning.

## Conclusion

The rapidly expanding genetic landscape of pituitary adenomas offers the potential for gene-tailored assessment and management strategies for patients and their families. In familial acromegaly and pituitary gigantism, genetic causes have been implicated and sometimes portend more aggressive disease and treatment resistance. Increasing access to genetic testing—particularly NGS platforms permitting testing of multiple genes simultaneously—should maximize detection of heritable familial acromegaly and pituitary gigantism, to the benefit of patients and the advancement in our knowledge of the genetic pathogenesis of pituitary adenomas.

## Data Availability

Data sharing is not applicable to this article as no datasets were generated or analyzed during the present study.

## Abbreviations

AHR: aryl hydrocarbon receptor
AIP: aryl hydrocarbon receptor–interacting protein
cAMP: cyclic adenosine monophosphate
CGH: comparative genomic hybridization
CMA: chromosome microarray
FIPA: familial isolated pituitary adenoma
GH: growth hormone
GHRH: growth hormone-releasing hormone
GPCR: G protein-coupled receptor
IGF-I: insulin-like growth factor 1
LOF: loss of function
LOH: loss of heterozygosity
MAS: McCune-Albright syndrome
MEN: multiple endocrine neoplasia
MRI: magnetic resonance imaging
NF1: neurofibromin 1
NGS: next-generation sequencing
PRKACB: protein kinase cAMP-activated catalytic subunit beta
SRL: somatostatin receptor ligand
TAD: topologically associating domain
VUS: variant of uncertain significance
X-LAG: X-linked acrogigantism
